# Vitamin D – a forgotten preventive agent against caries? A narrative review

**DOI:** 10.1007/s00784-025-06605-w

**Published:** 2025-12-01

**Authors:** Julia Grundmann, Susann Hertel, Christian Hannig, Johan P. Woelber

**Affiliations:** Faculty of Medicine Carl Gustav Carus, Policlinic of Operative Dentistry, Periodontology, and Pediatric Dentistry, TU Dresden, Fetscherstrasse 74, 01307 Dresden, Germany

**Keywords:** Vitamin d, Caries, Prevention, Demineralization, Remineralization

## Abstract

**Objectives:**

Vitamin D has been extensively studied in dentistry in the modern past. However, its impact on caries progression and arrest remains unclear. This narrative review aims to provide a critical overview of the relationship between vitamin D and caries, with a focus on the historical development of the topic, current scientific understanding, and potential future perspectives.

**Materials and methods:**

A literature review and screening of historical periodicals was performed to outline the role of vitamin D in caries prevention in the past and in the future. Articles focusing on the historic use of vitamin D in cariology and vitamin D in cariology were included and evaluated.

**Results:**

Literature showed that vitamin D was described as one of the central caries preventive agents in the 1930s and 1940s until systemic and topical fluoridation became more prominent. Possible mechanisms described by current science include effects on tooth development and immune modulation via saliva.

**Conclusions:**

The emergence of fluoride as the leading preventive measure in cariology shifted the focus away from vitamin D research. However, recent studies have renewed interest in the potential causal role of vitamin D in caries prevention.

**Clinical relevance:**

Further research is needed to examine the role of vitamin D in the proteome of saliva, pellicle and initial caries lesions, as well as to visualize its presumed arresting effect at the ultrastructural level. Additionally, prospective studies on the influence of vitamin D on caries progression in permanent teeth are necessary.

## Introduction

 Despite the availability of fluoride and oral hygiene programs, caries remains the most prevalent disease worldwide [1, 2]. Additionally, vitamin D deficiency affects 30–60% of children and adults globally [3]. Sugar consumption remains excessively high and continues to rise, with current intake levels reaching 126 g/d in the United States [4] and 91 g/day in Germany [5], far exceeding the recommended 25 g/d (equaling 5% of daily energy intake) [6]. These aspects highlight the persistent need for effective caries prevention strategies. While reducing sugar intake would address a causal factor, the precise role of vitamin D in caries arrest, despite existing evidence, remains largely unclear. The effects of vitamin D on the caries process seem to extend further than previously researched and the role that vitamin D may play in caries prevention appears to be greater than previously assumed.

This review provides a historical analysis of vitamin D research in cariology and its use as a prophylactic agent against rickets. Historical data from Germany and further European countries indicate that after the introduction of vitamin D prophylaxis in the late 1920 s, there was a decline in rickets [7]. Despite relatively stable sugar consumption, there was furthermore a decline in caries in the 1930 s and 1940 s, particularly in children [8–10]. Additionally, before the introduction of topical fluoride application, vitamin D supplementation was recommended after extensive restorative dental procedures as subsequent treatment [11].

Interestingly, hypotheses regarding the interaction of vitamin D and the caries process proposed over 100 years ago, were remarkably close to current assumptions. Therefore, revisiting the relationship between vitamin D and caries is necessary. The global rise of fluoride as the primary caries-preventive agent, despite substantial evidence supporting vitamin D, resulted in vitamin D being largely overlooked as a preventive agent in dentistry over the past eight decades.

The aim of this review is to provide an overview of the historical use of vitamin D in caries prevention and to expand upon new, innovative hypotheses based on past and current findings regarding vitamin D in cariology.

## Methods

The review was based on a combined electronic (PubMed) and analog (Saxon State and University Library (SLUB) Dresden, German National Library (DNB) Leipzig) literature search. Articles focusing on the historic use of vitamin D in cariology and vitamin D in cariology were included and evaluated. The reference sections of the included papers were manually screened for additional papers. English and German literature published from 1912 to 2025 was considered. Historical data was compiled and structured chronologically. In total, 18 historical and 29 recent studies or articles on vitamin D and dental caries were included in the analysis.

### Vitamin D – The sunshine vitamin

Vitamin D was discovered through the search for a cure for rickets, first mentioned in the 17th century, which we now understand as a disease caused by vitamin D deficiency [12–14]. Rickets is primarily understood as a bone disease, but it also has dental consequences, impacting the development of the dentition and teeth [15].

Alfred Windaus was awarded the Nobel Prize in 1928 for his work on the isolation and structural elucidation of vitamin D_2_ by ultraviolet radiation of ergosterol [16]. His findings were quickly translated into clinical medicine, as the mechanism of Windaus provided the foundation for the development of the vitamin D supplement Vigantol, which was introduced by the German pharmaceutic company Merck in 1927 and remained in production to this day.

Today, vitamin D is understood as a group of fat-soluble vitamins, steroids on a chemical level. Vitamins are typically defined as substances that cannot be synthesized by the body and must be obtained through diet. However, vitamin D precursors are produced within the body through the influence of UVB radiation and heat in the skin. Thus, vitamin D is not a true vitamin but rather functions as a (pro)hormone, with its synthesis being activated by sun exposure.

**Fig. 1:**
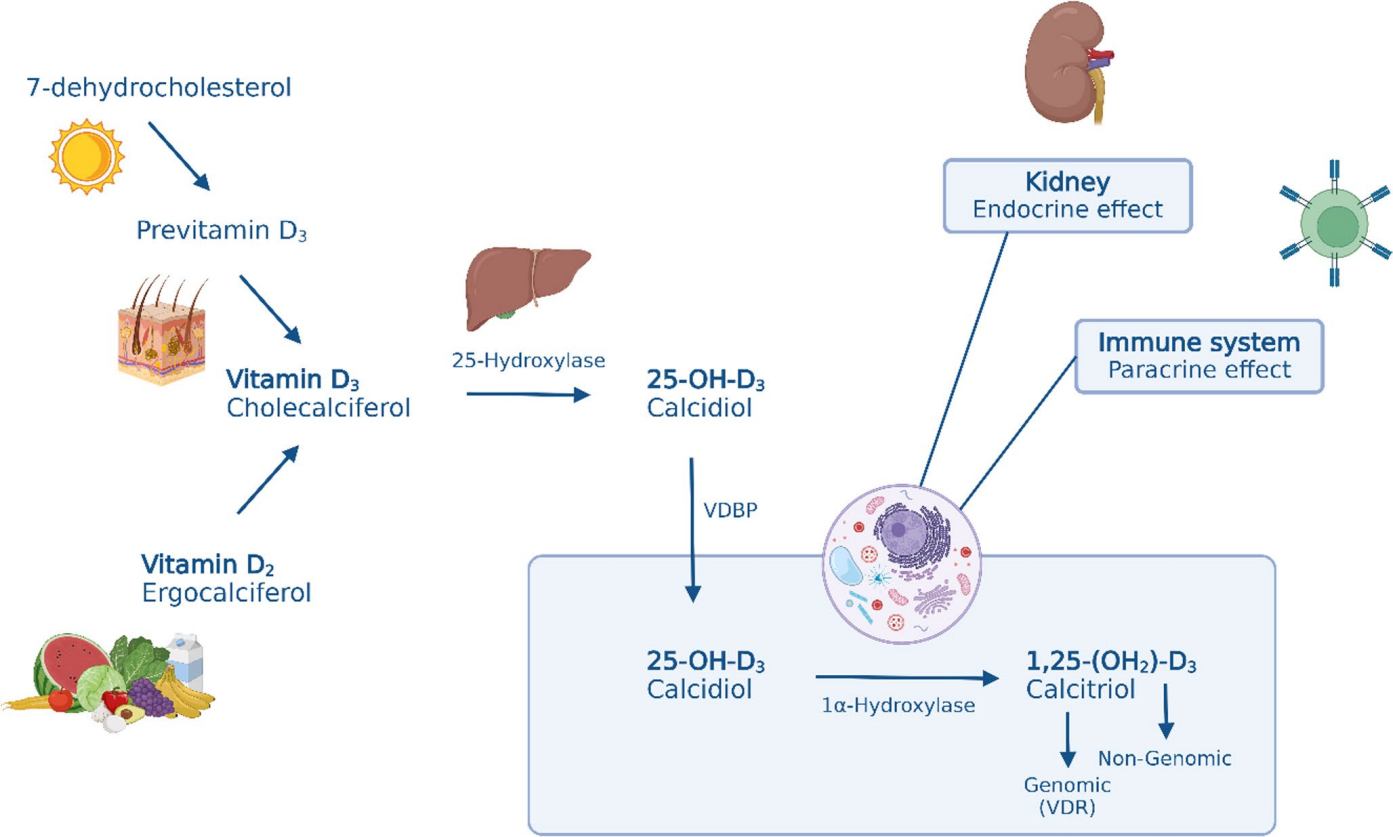
Pathway of vitamin D metabolism and its biological effects: Vitamin D3 is synthesized in the skin from 7-dehydrocholesterol under UVB exposure or obtained through dietary intake as Vitamin D2. Both undergo metabolic conversion in the body, primarily through the liver and kidneys, where they are transformed from their prohormone forms into active hormones via hydroxylation. In the liver, it is converted to 25-OH-D3 (calcidiol) by 25-hydroxylase, then further metabolized in the kidney by 1α-hydroxylase to its active form, 1,25-(OH)2D3 (calcitriol). Calcitriol exerts endocrine effects via the kidney and paracrine effects on the immune system. Calcitriol acts through genomic (VDR) and non-genomic pathways at target cells

Ergocalciferol (vitamin D_2_, derived from plant sources) and cholecalciferol (vitamin D_3_, derived from animal sources) are the two main forms of vitamin D (Fig. 1). About 80% of vitamin D is generated through skin synthesis, where 7-dehydrocholesterol is converted into previtamin D_3_ under the influence of UVB radiation. This previtamin D_3_ is then isomerized into vitamin D_3_ (cholecalciferol) by heat. The remaining 20% are obtained through dietary intake as vitamin D_2_ (ergocalciferol), including food sources and/or supplements. By the addition of a hydroxyl group, cholecalciferol is converted into calcidiol (25-OH-D_3_) in the liver, as shown in Fig. 1. The fat-soluble calcidiol is then transported in the aqueous bloodstream, whereas the vitamin D_3_ metabolites are mainly bound to vitamin D-binding protein (VDBP) and albumin and the vitamin D_2_ metabolites are transported via liprotein particles [17, 18]. Once calcidiol reaches the target cell, it is taken up and undergoes a second hydroxylation, primarily in the kidneys, to become calcitriol (1,25-(OH_2_)D_3_), the active form of vitamin D. This active form then exerts its effects by binding to the vitamin D receptor (VDR) or activating non-genomic intracellular signaling pathways [3, 13, 18–20].

The primary function of vitamin D, maintaining calcium homeostasis, is achieved through its endocrine role, in coordination with hormones such as parathyroid hormone (PTH), with the kidneys, intestine and the bones serving as key organs. Its importance lies in the regulation of calcium and phosphate metabolism within a narrow range. This endocrine regulation ensures the balance of calcium level in the blood around 1 mmol ionized calcium or 10 mg/100 ml of total calcium, which, from an evolutionary perspective, is a priority for physiological stability. Beyond its endocrine effects, vitamin D also exerts paracrine actions, particularly on immune cells. These paracrine effects are important in modulating immune responses, including the regulation of inflammation and pathogen defense. These dual mechanisms allow vitamin D to play a versatile role in maintaining health at both the cellular and systemic levels. Therefore, vitamin D supports healthy bone tissue, enables muscle contraction, contributes to multiple immune functions and ensures normal cellular activity throughout the body [14].

Today, vitamin D deficiency represents a global health issue, affecting 30–60% of children and adults worldwide [3]. Contributing factors include insufficient skin synthesis of vitamin D_3_ due to excessive use of sunscreen, air pollution, a predominantly indoor lifestyle in Western societies, diminished synthesis capacity with aging, and inadequate dietary intake of vitamin D_2_ [3, 20].

## Caries as a multifactorial disease

Caries is a disease that leads to the destruction of dental hard tissues. It is biofilm-dependent, multifactorial, and influenced by the patient’s dietary behavior. Here, the cariogenity of the diet, which is determined by the proportion of highly processed and rapidly fermentable carbohydrates, as well as the frequency of sugar intake, is of particular importance. The key event is the alteration of the sensitive ecosystem of the oral cavity, leading to the dominance of pathogenic bacteria in a former physiologically balanced biofilm [21, 22].

**Fig. 2:**
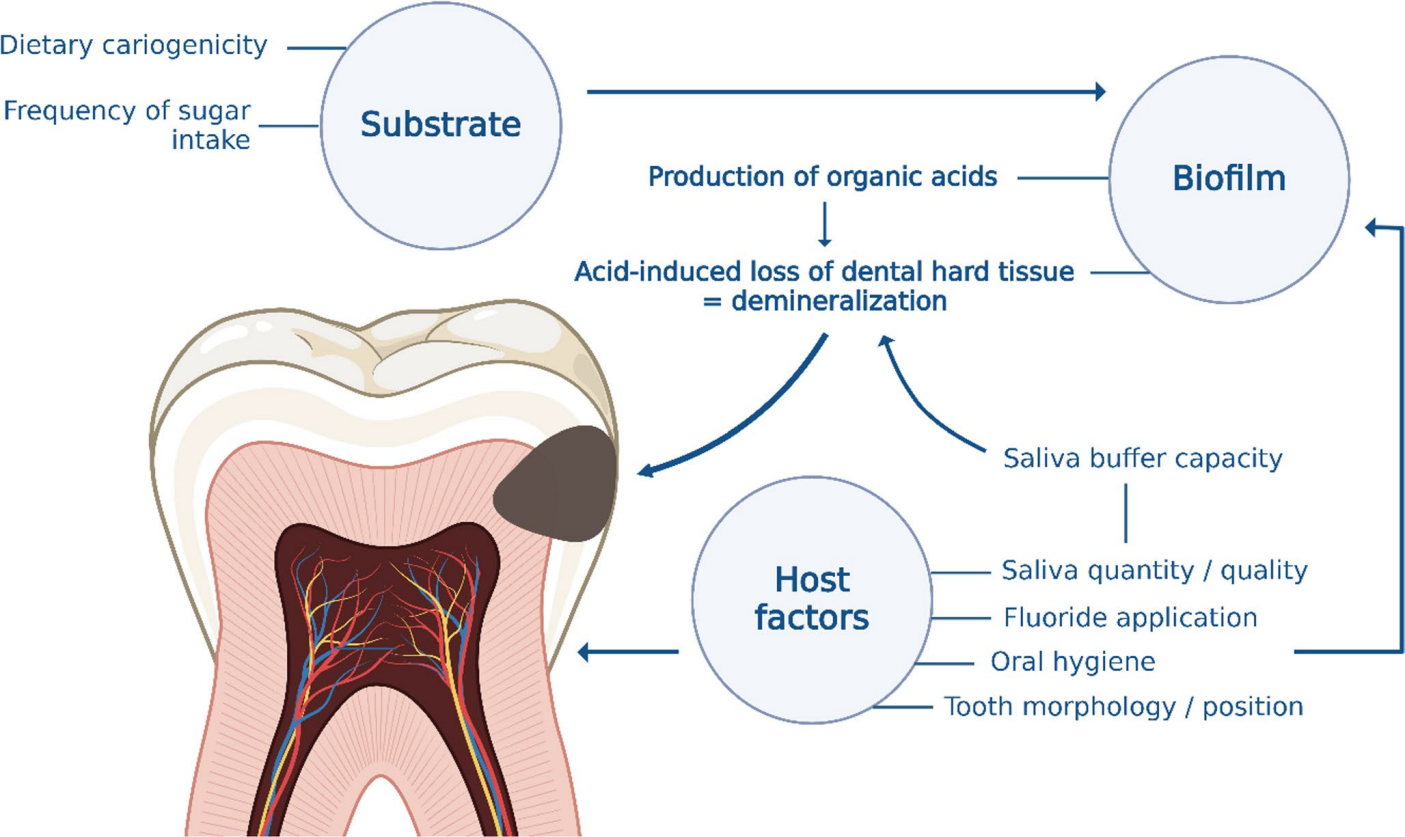
Caries is a multifactorial disease. The interplay between the biofilm at the tooth surface, substrate through dietary intake, and host factors influences caries development. Biofilm formation involves organized microbial communities adhered to the enamel surface, producing organic acids that lead to demineralization. Substrate, particularly dietary sugar intake, fuels this process and is the primary factor in promoting the progression of caries. Malnutrition, along with additional host factors such as saliva quality and quantity, oral hygiene practices, fluoride application, and tooth morphology, modulate caries predisposition. Saliva plays a key role in the physiological defense against caries by neutralizing acids through buffering and providing immunomodulatory protection

As shown in in Fig. 2, the metabolism of fermentable carbohydrates by bacteria in the biofilm on tooth surfaces leads to acid production. The subsequent drop in pH at the tooth surface leads to the demineralization of tooth enamel, specifically the dissolution of calcium and phosphate ions from the enamel surface. This subclinical stage of demineralization is constantly remineralized by the buffering capacity and ion reservoir of saliva [22]. Saliva is an ion-filled, calcium-rich aqueous solution. Calcium concentration ranges from 0.86 ± 0.46 mmol/l in unstimulated saliva to 1.11 ± 0.21 in stimulated saliva [23]. When the buffering systems of saliva are overused, an imbalance occurs in the believed de- and remineralization process [24]. As mineral loss progresses, an initial carious lesion becomes visible as a “white spot” on the tooth surface. The integrity of the enamel surface initially remains intact, although the surface appears rough when the lesion is active [22].

Within this concept of progression and arrest or de- and remineralization it can be asked if remineralization is even possible? This question had already been raised by Ernst Schmitz, who critically examined the experiments of Pickerill (1913) and Head (1910). In 1862, Hoppe-Seyler discovered that saliva must “harden” the teeth after eruption. Based on the inability to explain an enamel metabolism, it was concluded at the time that this inability served as evidence for an external supply, specifically through saliva. Notably, Head’s experiments lacked proof that calcium had dissolved and, conversely, that calcium had been redeposited following treatment with saliva. Back then, Head hypothesized that slowly migrating molecules could pose a barrier to subsequent molecules. Head’s initial uncertainty about his own experiments (“a sort of recrystallization”) has gradually faded, consolidating the theory of remineralization and demineralization, which remains widely accepted textbook knowledge to this day [21, 22, 25, 26].

## The role of vitamin D in caries prevention in the past

After the introduction of Vigantol for the prevention of rickets, a study by Mellanby and Pattison in 1928 [27] gained attention regarding the effects of vitamin D on the progression of caries in children. Beforehand, two studies were published in 1924 and 1926 in which the influence of diet on caries progression was examined in children with caries. In the first study in 1924 [28], the amount of calcifying foods like milk, eggs and cod-liver oil as well as oatmeal was varied. In the second study in 1926, the amount of protein, carbohydrate and fat intake remained stable between the groups [29], only the amount of fat-soluble vitamins and the amount of oatmeal was varied. Both studies showed that diets with a high calcium content limited the initiation and spread of caries, whereas diets of low vitamin content and furthermore containing oatmeal showed no such effect. Mellanby and Pattison hypothesized that vitamin D was responsible for the calcification of teeth and the hardening of the caries lesions. Therefore, in the study of 1928, only the amount of vitamin D was varied between the groups. The children were given additional vitamin D (irradiated ergosterol in the form of radiostol) over a period of 28 weeks in varying concentrations of 1 ml, 2 ml and 4 ml, additionally to a diet that contained milk, butter, sugar, jam, potatoes, meat and fruit. This resulted in an average “hardening” of caries in 3.9 teeth per child. The average number of teeth per child showing initiation or spread of caries was 1.0 per child. In comparison, the group of children of 1926 that did receive abundant fat-soluble vitamins showed a “hardening” of 2.0 caries lesions and initiation or spread of caries of 1.8 per child. The group of children in 1926 that did receive nearly no fat-soluble vitamins and oatmeal instead of bread showed “hardening” of caries lesions of less than 0.1 per child and initiation or spread of caries of 5.8 per child. The authors were thus able to demonstrate that existing caries lesions could be arrested and the formation of new lesions prevented. This means that with a nearly equal caloric intake, a diet which also included sugar but differed in vitamin D content, the group with vitamin D supplementation showed the highest rate of “healed”, nowadays called “arrested”, caries, showing a potential association between vitamin D and caries arrest. The study was conducted on children but also mentioned the case of a 22-year-old in whom similar effects were observed in the wisdom teeth. Furthermore, it has to be noted that the children in 1924 and 1926 had an average age of 9 years, whereas in the third study of 1928, the average age was 5.4 years [27]. It must be noted that the three studies discussed above were observational case series without randomization or control groups. In another study, Pattison found that the calcium content of saliva appeared to increase during vitamin D supplementation, suggesting that part of the vitamin D effect might be mediated by the increased calcium content in the saliva [26]. This promising study was one of the first of its kind and laid the foundation for the initiation of multiple studies. As a consequence, the American Dental Association (ADA) significantly advanced research on vitamin D and caries from 1930 onwards, leading to further studies to be conducted on healthy children as well [30].

In Germany, the rickets prophylaxis with vitamin D was introduced into infant care in 1926 at the School Dental Clinic in Bonn by Kantorowicz, a dentist who significantly contributed to the implementation of school and youth dental care in Germany. After only 2 years, less than 1% of children under the age of one year showed signs of rickets, making Bonn the first rickets-free city worldwide. Throughout the 1930 s, Vigantol was extensively promoted in German journals and used for rickets prevention. According to the initiator Kantorowicz, rickets prophylaxis was the most effective means of reducing caries. In 1928, he wrote that the extent of rickets prophylaxis on dental health could only be guessed at, referring to the change in dental constitution associated with the eradication of rickets. Within just 5 years, caries rates in children decreased by 60%. The extraction of primary first molars decreased by 63%, and the extraction of primary second molars by 45% [7, 9].

Starting in 1933, cod liver oil and Vigantol were given to children in need at the infant home in Dortmund, leading to more standardized tooth eruption times compared to children without rickets prophylaxis. Here, vitamin D was combined with vitamin A (Reher, 1936). Summarized, in the 1930 s, rickets prevention in Germany was mainly carried out with Vigantol and cod liver oil, accompanied by nutritional prophylaxis against caries. It is important to note that vitamin D has never been used solely as a caries-preventive agent. Rather, it supported normal tooth development through rickets prevention, which consequently resulted in caries prevention as a side effect. Interestingly, it is mentioned in a post-war summary on the current state of prophylaxis in dentistry, that a high-dose vitamin A, C, and D therapy following filling treatment is recommended [11].

Remarkable experiences with Vigantol for rickets prophylaxis in Germany became apparent about 25 years later, when the epidemiology of dental caries could be retrospectively assessed (pre-war and post-war periods). It must be noted here as well that Kantorowicz’ historical epidemiological study relies on observational cross-sectional data without randomization or control groups. A phenomenon known as the “German caries miracle”, which refered to a 75% reduction in caries prevalence among 13–14 year-olds (limited to the birth cohorts of 1930–1943), and furthermore accompanied by a 90% reduction in caries prevalence in Berlin among 6–7-year-olds (birth cohorts 1928–1942). This phenomenon and the furthermore reduction in observed hypoplasias has been explained by two main hypotheses:

1. The use of vitamin D for rickets prophylaxis in Germany from 1926 onwards, which secondarily had a significant positive effect on tooth structure and consequently dental health.
2. The drastic reduction of sugar consumption during the war years. However, it is important to critically note that pre-war cohorts (1930–39), without sugar restrictions, already showed significantly lower rates of caries [7, 9].

Kantorowicz dismissed the sugar theory as primary causes, noting that the per capita sugar consumption remained stable between 1930 and 1943 at approximately 14–15 kg annually, comparable to sugar consumption levels at the turn of the century when caries incidence was four times higher. Instead, Kantorowicz proposed the constitutional theory, suggesting that the absence of rickets in childhood facilitated the development of healthy, resilient enamel [7, 9, 30].

Interestingly, researchers from other countries who also investigated the effects of vitamin D reported similar results: a decline in caries incidence between the world wars, specifically from 1930 to 1940, followed by a subsequent rise. Mellanby suggested that no definitive cause for the reduction in caries frequency in London could be identified; however, dietary changes during the war years, particularly the rationing of sugar, were likely contributing factors [31]. Epidemiological studies and case series from Norway report similar findings: Alexander reported from Norway that before and after the war, cod liver oil was only given to children to a limited extent. However, during the war years, especially from 1942 onward, milk and cod liver oil was distributed to children in schools to ensure their vitamin intake. There was a decline in caries from 1942 onward in Norway, whereas after the war, from 1947 onward, caries reappeared in schoolchildren. Alexander assumed that the reduced sugar consumption due to rationing during the war years was the reason for this decline [8]. This is in line with the findings of Toverud, who reported on surveys conducted by the pediatric department in Norway during the war among schoolchildren. There were more caries-free children during the war, with a 36% decline in caries (in terms of affected teeth) and in terms of affected surfaces a reduction of 82% in first graders as well as 66% in 14-year-olds between 1939 and 1945. This decline was also observed in preschool children. Toverud reported on measures to ensure a calcium- and vitamin-rich diet for pregnant women, breastfeeding mothers, and schoolchildren. Sugar consumption was rationed to 30 g per day and almost completely disappeared from the market in the final years of the war. Therefore, he concluded, a correlation between the decline in caries and the reduction in sugar consumption was observed [10].

Furthermore, studies conducted between the world wars in the USA, UK, and Canada, involving 24 controlled clinical trials (CCTs) on 2,827 children aged 2 to 16 years, demonstrated up to a 47% reduction in caries incidence with vitamin D supplementation [32]. It is important to note that all CCTs were conducted during a time of different dietary practices, varying sun exposure, and minimal fluoride use. Hujoel also emphasized the significant marketing efforts for vitamin D during that period, a phenomenon reflected in German journals of the time, and notes that conflicts of interest can no longer be fully separated in retrospect [32]. During the interwar period, there was a ‘sunshine era’ where sunlight was considered essential for health, until the discovery of skin cancer later led to reduced sun exposure [12].

As outlined by Hujoel in a historic review about the rise of fluorides in dentistry, the influence of vitamin D on the development of caries was considered proven by many dentists in the United States. Beginning with an ADA statement in 1930, vitamin D was recognized in caries prevention for 15 years in the United States. In the 1940 s, caries prevalence was 96% among 15-year-olds, combined with a prevalence of 47% for rickets in children. In 1944, vitamin D still was the only preventive agent against caries recognized by the ADA since toothbrushes, toothpastes, and fluorides had not yet been accepted. However, in 1945, the ADA published a brief abstract, which led to a shift in preventive strategies, as vitamin D was no longer recommended as a caries prophylactic agent despite multiple studies indicating its effect on the caries process, as documented by the National Academy of Sciences. Subsequently, topical fluoride application and water fluoridation replaced vitamin D as primary preventive measures. Public health policies at the time prioritized fluoride due to emerging strong evidence supporting its effectiveness [30]. It should also be noted that vitamin D received less attention potentially due to limited mechanistic understanding and changing scientific priorities. At this time, the metabolism of vitamin D and its complete mechanisms were not fully understood, as research on these aspects only intensified in the 1960 s [14].

Following these developments, fluorides were mentioned in Germany in 1948 in the *Deutsche Zahnärztliche Zeitung (German Dental Journal)*, where research from the United States and England was reported [33, 34]. As the efficiency of fluoride prophylaxis gained widespread recognition, particularly starting in the United States, advertisements for Vigantol disappeared from dental journals. An analysis of the periodicals from that era revealed that it was briefly replaced by a new preparation called “Paradenyl” a hormone-vitamin combination paired with supplementary electrolytes, before being replaced by the first fluoridated toothpaste, “BioXFluor”, in the 1950s.

Baume [33, 34] frequently reported on developments in fluoride research from American conferences in German dental journals. He noted that the German physician Erhardt was the first to recommend fluoride tablets in 1874, although current fluoride prophylaxis has now become the initiative of the U.S. Public Health Service. From 1939 to 1941, the first mass prophylaxis trials were conducted in the U.S. as epidemiological comparison studies between low-fluoride and high-fluoride areas, demonstrating that water fluoridation was associated with a 60% reduction in caries incidence. Back then, the extent of caries was measured using the DMFT index. However, Baume pointed out statistical errors: first, only cavitated caries was observed, as no X-rays were used to detect approximal caries. Second, he criticized the results as the reported reduction rates (up to 75%) were difficult to verify due to the use of inconsistent reference values for caries reduction and progression. The action of fluorides, specifically the formation of fluorapatite, was initially demonstrated through experiments using powdered enamel. However, Baume reported that in-vivo studies in the U.S. have not provided conclusive evidence. Baume emphasized that caries is not a fluoride deficiency disease. While fluoride-treated teeth are more resistant to caries, the underlying mechanism remains unclear [33, 34].

German dentists were initially critical of the rise of fluoride use, as the previous described significant decline in caries in Germany was observed even without the implementation of mass fluoride prophylaxis [7, 9, 35]. It should also be noted that the “German caries miracle” occurred without any influence from systemic water fluoridation or local fluoride application [7].

Despite a massive decline in tooth decay in Western Europe and the USA, caries remains the most common disease worldwide [1, 2]. Current prevention strategies tend to focus more on symptomatic treatments, such as the use of fluorides and plaque control, while nutritional counseling, as a causal therapy, often receives little attention. In addition to topical fluoride application, dentists could also prescribe vitamin D, given its widespread deficiency and the general health benefits of adequate supplementation [30, 36]. In summary, following the introduction of fluorides, the role of vitamin D in caries prevention received less attention, while recent years have seen a renewed interest in vitamin D research within cariology.

## Current hypotheses on the impact of vitamin D on caries mechanisms

Current hypotheses on caries etiology do not extend beyond what was already proposed in the early years of vitamin D supplementation for caries prophylaxis. While early hypotheses on caries prevention through vitamin D supplementation emphasized its contribution to enamel mineralization, they also proposed several additional mechanisms: improved tooth development, a fluoride-like topical effect, alterations in the quantity or biochemical composition of saliva, and immunological modulation of caries activity, a forward-thinking idea for the 1930 s [32, 36].

1. *The interplay between vitamin D and caries risk (DMFT)*.

A study by Asante et al. demonstrated a dose-dependent inverse relationship between serum vitamin D levels and DMFT scores, suggesting that lower vitamin D levels are associated with higher caries prevalence [37]. For deciduous teeth, Bumbu et al. noted a probable association between vitamin D deficiency and increased caries risk, though some studies in this systematic review reported divergent results, emphasizing the multifactorial nature of caries development [38]. For permanent teeth, Buzatu et al. also reported variable associations, suggesting a protective role for adequate vitamin D levels and increased risk with deficiency, albeit based on cross-sectional data [39]. Hung et al. (2024) found that severe vitamin D deficiency correlated with a 1.13 higher DMFT score in older adults [40], while a U.S. population-based study supported the association between vitamin D insufficiency and higher caries risk [41].

Despite these findings, the evidence on vitamin D’s role in oral health remains inconsistent. While some studies support a link between vitamin D deficiency and higher caries prevalence, others find no significant relationship [42].

Durá-Travé and Gallinas-Victoriano proposed three potential mechanisms by which vitamin D might influence the caries process: prenatal effects on tooth mineralization, its role in antimicrobial peptides, and its impact on salivary flow rate and salivary calcium content [43]. While animal studies suggest a link between vitamin D and reduced salivary calcium [44, 45], human data on this mechanism remain unavailable. Table 1 provides an overview of studies published in the last 10–15 years investigating the relationship between vitamin D and caries prevalence.

**Table 1:** Overview of studies on vitamin D levels and dental caries

Author, Year	Study Design	Age/Population	Vitamin D Measurement	Outcome	Main Finding
Grant, 2011 [Review]	Narrative review	General population	N/A	Dental caries	Suggests UVB-induced vitamin D may reduce caries risk through immune modulation and enamel effects.
Asante et al., 2024	Cross-sectional observational	Adults and children (HUNT study)	Serum 25(OH)D	Caries prevalence and periodontitis	Dose-dependent inverse association between serum vitamin D and caries/periodontitis; evidence mostly cross-sectional.
Bumbu et al., 2023 [Systematic Review]	Systematic review	Children (deciduous teeth)	Mostly serum 25(OH)D	Caries susceptibility	Probable association between low vitamin D and increased caries risk; mixed results in included studies.
Buzatu et al., 2024 [Systematic Review]	Systematic review	Children and adolescents (permanent teeth)	Serum 25(OH)D	Caries prevalence	Variable associations; some evidence for protective role of adequate vitamin D, but inconsistent overall.
Hung et al., 2024	Cross-sectional observational	Older adults (> 60 years)	Serum 25(OH)D	Caries prevalence	Severe vitamin D deficiency correlated with higher DMFT scores; supports association in older adults.
Padmanabhan et al., 2024	Cross-sectional observational	Children	Salivary vitamin D and vitamin C	Caries prevalence	Found association between lower salivary vitamin D levels and higher caries prevalence.
Tapalaga et al., 2023 [Systematic Review]	Systematic review	Pregnant women/infants	Serum 25(OH)D	Enamel defects, tooth erosion	Prenatal vitamin D deficiency linked to enamel defects; uncertain impact on early childhood caries (ECC).
Durá-Travé & Gallinas-Victoriano, 2024	Narrative review	Children	Various	Dental caries	Review highlighting vitamin D deficiency as a risk factor for dental caries, emphasizing multifactorial etiology.
Botelho et al., 2020 [Comprehensive Review]	Review	General oral health	Various	Oral health parameters	Comprehensive review; vitamin D deficiency linked with poor oral health including caries; mechanistic insights included.
Singleton et al., 2022	Observational (program evaluation)	Alaska Native infants	Vitamin D supplementation records	Early childhood caries	Prenatal supplementation reduced vitamin D deficiency and ECC prevalence in high-risk population.
Schroth et al., 2020	Observational/Cohort	Infants	Prenatal vitamin D supplementation	Dental caries incidence	Prenatal vitamin D supplementation associated with reduced caries in infants.
Pratyusha et al., 2021	Cross-sectional observational	Children (3–11 years)	Serum vitamin D, salivary calcium, phosphorus	Caries prevalence	Vitamin D deficiency correlated with lower salivary calcium and higher caries prevalence.

2. *Vitamin D deficiency in pregnancy: implications for the child’s caries risk*.

The relationship between vitamin D and dental health often emphasizes the pre-eruptive effects of vitamin D on the mineralization of dental hard tissues. Like nearly all human cells, ameloblasts and odontoblasts also express a vitamin D receptor (VDR) [43]. Maternal vitamin D deficiency during pregnancy has been linked to structural enamel disorders, such as amelogenesis imperfecta, dentinogenesis imperfecta, and enamel hypoplasia [46, 47]. An Alaskan study showed that prenatal women with insufficient vitamin D serum level in late pregnancy hat offspring with higher dmft scores at the age of 12–35 month than prenatal women with sufficient vitamin D levels (Singleton et al., 2022). Schroth et al. did not find a relation between the prevalence of Early Childhood Caries (ECC) and the maternal vitamin D level during pregnancy [48]. While vitamin D appears to influence the pre-eruptive mineralization of primary teeth, its potential remineralizing effect on permanent teeth remains insufficiently demonstrated [42].

Tooth mineralization depends on adequate serum levels of vitamin D, calcium, and phosphate [49]. Nørrisgaard demonstrated that high-dose vitamin D supplementation during pregnancy reduced the risk of enamel defects in children aged 6 years by approximately 50% [50]. Kühnisch et al. investigated the relationship between serum vitamin D levels and the risk of Molar-Incisor Hypomineralization (MIH), finding that low vitamin D levels were associated with a higher risk of MIH in 10-year-olds [51]. In contrast, van der Tas et al. concluded that vitamin D levels during the prenatal and early postnatal periods, as well as at age 6, were not linked to an increased risk of MIH [52].

3. *The impact of vitamin D on saliva quality*.

Padmanabhan et al. found significantly lower levels of vitamins D and C in the saliva of caries-active children compared to caries-free children [41]. Pratyusha et al. showed that a high prevalence of dental caries is associated with low serum vitamin D levels, reduced salivary calcium, and increased salivary phosphate. Additionally, an inverse relationship was observed between the number of decayed teeth and both salivary calcium and serum vitamin D levels [53].

The salivary glands also express a vitamin D receptor (VDR), indicating that vitamin D deficiency might reduce salivary flow rate. It is assumed that the decreased mineral content in saliva may result in insufficient remineralization even though a reduction in mineral content has not yet been definitively proven [43]. The assumption that vitamin D deficiency reduces salivary flow rate and salivary calcium content is based on studies conducted in rats. It was shown that parotid gland secretion is decreased in rats deprived of vitamin D. It is important to note the rats that exhibited a 65% reduction in parotid gland salivary flow were not only fed a vitamin D-deficient diet but also born to mothers with vitamin D deficiency. In contrast, the rats that were only fed a vitamin D-deficient diet maintained a stable parotid gland salivary flow, despite the lack of vitamin D in their diet. The authors concluded that the availability of calcium in the extracellular fluid is independent of vitamin D, whereas the secretion of electrolytes and water appears to be directly dependent on vitamin D. Even the calcium concentration in parotid gland saliva remained stable in vitamin D-deficient rats. Therefore, vitamin D might be essential for the synthesis of proteins that utilize extracellular calcium in the secretion processes of the parotid gland [44, 45].

4. *Vitamin D and its role in innate immune defense (AMP)*.

Saliva is an essential component of the innate immune system. Key components of saliva include the cathelicidin peptide LL-37, alpha-defensins, beta-defensins, histatin, and statherin. Additionally, major salivary glycoproteins such as mucins, proline-rich proteins, and immunoglobulins, along with minor salivary glycoproteins such as agglutinin, lactoferrin, cystatin, and lysozyme, contribute to oral defense mechanisms. Antimicrobial peptides like cathelicidin LL-37 are host defense peptides that play a crucial role in innate immunity of saliva. The synthesis of cathelicidin LL-37, which is an antimicrobial peptide (AMP), is activated by the vitamin D–VDR complex [54, 55]. Similarly, the production of beta2-defensin is also regulated by this complex [43]. Cathelicidin LL-37 is part of salivary defense proteins and primarily originates from neutrophil leukocytes [56].

Cathelicidin-LL-37 is the fourth most abundant AMP in the human body and the third most prevalent in saliva [57]. Cathelicidin LL-37 exhibits antimicrobial properties and anti-endotoxin activity. Due to its cationic and amphiphilic nature, it depolarizes and disrupts the cellular membrane of pathogens [55].

According to Tokajuk et al., LL-37 plays a key role in controlling oral biofilm. It is hypothesized that specific AMP cocktails serve as a physiological response against pathogens, representing an adaptive co-evolution between beneficial bacteria that induce AMPs to combat harmful bacteria [57]. Additionally, the response to pathogens appears to be oxygen-dependent. Facultatively anaerobic bacteria are less susceptible to LL-37 than obligate anaerobic bacteria [58].

He et al. conducted a 14-week vitamin D supplementation study in athletes that measured the levels of sIgA, lactoferrin, and cathelicidin. The results showed that cathelicidin levels increased in the vitamin D group but decreased in the placebo group. Lysozyme levels and salivary flow rates increased over time. The levels of sIgA and lysozyme increased, while lactoferrin levels remained stable in the vitamin D group. The study did not assess the caries status of the athletes [59].In correlation with caries prevalence, Tokajuk et al. suspect that individuals with low caries levels tend to have high LL-37 levels, although contradictory findings have also been reported [57].

Pellicle formation is another defense mechanism of saliva, although it serves as the initial site for bacterial adhesion. The acquired pellicle serves as a barrier that prevents demineralization to a limited extent. There are 68 universally pellicle proteins that are universally present, while the remaining proteins vary among individuals [60]. Furthermore, out of fifteen core proteins, eleven are involved in innate immune defense. Further research is needed to determine whether vitamin D has an impact on the variation of pellicle proteins or presumably caries infiltrating proteins. To the best of the authors’ knowledge, no studies have explored the influence of vitamin D on the pellicle proteome.

5. *The impact of vitamin D on the oral microbiome*.

The impact of vitamin D on the oral microbiome and its relation to the current ecological plaque hypothesis has not been thoroughly investigated. Charoenngam et al. reported that an increase in serum vitamin D levels is associated with a rise in beneficial bacteria and a reduction in pathogenic bacteria in the gut [61]. Similarly, Vigors et al. demonstrated that vitamin D supplementation affects the oral microbiome in dairy calves, noting the abundances of *Streptococcus* and *Actinobacillus* after seven months of vitamin D supplementation. The mechanism underlying the influence on the oral microbiome remains unclear [62].

6. *The influence of vitamin D on remineralization of caries lesions*.

The impact of vitamin D on remineralization of caries lesions remains uncertain. Al-Jubori et al. demonstrated that saliva from patients receiving vitamin D supplements exhibited a higher remineralizing effect on artificially created ex vivo initial lesions. This effect was attributed to an increased calcium and phosphate content within the carious lesion, along with enhanced microhardness [63]. As of today, remineralization of a caries lesion through vitamin D, specifically through mineral deposition in the caries lesion, has not been visualized or conclusively proven.

**Table 2:** Overview of current hypotheses on the role of vitamin D in caries prevention

Hypothesis	Details
Vitamin D deficiency is associated with increased caries prevalence	Cross-sectional and observational studies suggest that low serum vitamin D levels correlate with higher dmft indices, particularly in children.
Maternal vitamin D deficiency may impair enamel formation in offspring	Vitamin D deficiency during pregnancy has been linked to structural enamel defects in primary teeth; however, the association with early childhood caries (ECC) remains inconclusive.
Vitamin D influences salivary composition	Vitamin D deficiency is associated with lower salivary vitamin D and calcium levels; its effect on salivary flow rate remains unclear.
Vitamin D modulates the innate immune defense in saliva	Vitamin D–VDR signaling regulates the synthesis of key antimicrobial peptides such as cathelicidin LL-37 and β-defensin 2, which play essential roles in the salivary innate immune system. LL-37 exhibits broad-spectrum antimicrobial and anti-endotoxin activity and may contribute to oral biofilm control. Vitamin D supplementation increases salivary LL-37 levels; however, the impact on caries risk remains inconclusive.
Potential influence of vitamin D on the oral microbiome	The impact of vitamin D on the composition or function of the oral microbiome has not yet been investigated in detail.
Vitamin D may support enamel remineralization	A potential role of vitamin D in promoting enamel remineralization has been suggested, but this effect has not yet been clearly demonstrated or visualized in experimental or clinical settings.

As summarized in Table 2, the majority of clinical studies focus on children, while a significant knowledge gap remains regarding the role of vitamin D in caries prevention within the adult population. However, topical effects on organic and inorganic interaction at the tooth surface and the influence on the oral microbiome have not yet been demonstrated.

## Innovative perspectives on the role of vitamin D in caries prevention

*“… and I would like to emphasize here*,* too*,* again that it is the enamel surface which is so important; it is at the very surface of the enamel that the battle is won or lost*,* …”* [64].

**Fig. 3:**
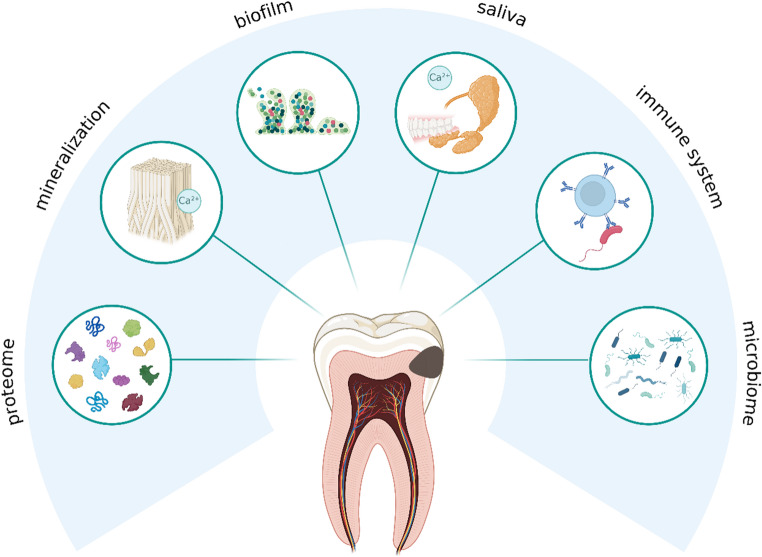
Presumed local effects of vitamin D in the oral cavity, especially in relation to caries: In addition to its pre-eruptive effect on enamel formation and mineralization, as well as its influence on the innate immunity of saliva, vitamin D may also affect the oral microbiome and biofilm formation. A topic that has not yet been investigated is its influence on the proteome of initial caries lesions, another potential target for vitamin D

Currently hypothesized mechanisms of vitamin D in caries pathogenesis should be considered as conceptual models. As outlined before, it appears to have an anti-cariogenic or cariostatic effect, but its exact mode of action remains unexplained. The common assumption is that vitamin D deficiency during pregnancy leads to poor mineralization of the children’s teeth, which increases susceptibility to caries. In fact, high-dose vitamin D supplementation during pregnancy results in a 50% reduced risk of developing enamel defects [50]. However, there is no correlation between maternal vitamin D intake and caries incidence in the deciduous dentition, which supports the idea of a predominantly local, topical effect occurring postnatally. Therefore, the influence of pre-eruptive enamel maturation on future caries risk should not be overestimated. Furthermore, to date, no prospective study has investigated the influence of vitamin D on permanent teeth. Conventional dental knowledge and practice are based on the theory that constant demineralization and remineralization processes occur at the tooth surface [65]. Current dental research challenges the existence of demineralization and remineralization in the traditional sense [60], since the interactions between organic and inorganic components at and beneath the enamel surface are not yet understood, despite their clinical relevance. This raises the question of whether there is a remineralization of caries lesions or rather an impregnation with proteins that hinder remineralization. Salivary proteins involved in pellicle formation may play a role in this inhibition [66]. If this is the case, it would further underscore the role of vitamin D as an immunomodulatory and anti-inflammatory hormone in caries prevention due to influencing the innate immune system of saliva. Vitamin D might support the pellicle formation or exert its effect via antimicrobial peptides in saliva. Considering this, all hypotheses suggesting that “vitamin D promotes remineralization” would need to be rejected. This also explains the uncertainty regarding the true effect of vitamin D on biofilm accumulation and caries progression. When it comes to the influence of vitamin D on saliva, more than salivary flow rate and salivary calcium content are of importance. The relationship between vitamin D and saliva extends to the salivary proteome, immunological parameters, and interactions with initial carious lesions, as shown in Fig. 3. Furthermore, the role of the pellicle and oral microbiome in caries progression remains a key factor. Despite effective oral hygiene, microbial adhesion continuously occurs [67]. Vitamin D may have a direct effect on cariogenic biofilm formation and its structural organization.

Possible mechanisms for the anti-cariogenic effects of vitamin D could involve the modulation of salivary and pellicle proteins rather than direct promotion of remineralization. Such modulation could affect protein-mediated processes associated with caries arrest, shifting the focus from strengthening dental hard tissues towards dynamic surface interactions. It can be concluded that the key question is how vitamin D interacts with organic and inorganic components (proteins and minerals) within and on white spot lesions.

## Future directions

Due to the widespread use of fluorides over the past decades, replicating study conditions from a century ago is no longer feasible. There is a need for studies assessing the influence of vitamin D on caries prevalence and incidence in children and vulnerable populations. Here, relevant confounding variables need to be taken into account, as the existing data still do not clarify whether and how vitamin D might influence caries development or arrest. Furthermore, there is a need for prospective longitudinal studies on caries arrest in adults with and without vitamin D deficiency to gain deeper insights into its therapeutic potential.

In the future, investigating whether a balanced vitamin D level could serve as a causal caries prevention could provide valuable insights into non-invasive dental care strategies. Key questions include whether vitamin D exerts its effects mainly through systemic pathways or via topical action, how it influences demineralization and remineralization processes, and whether it helps arrest initial carious lesions by affecting interactions between organic and inorganic components at the tooth surface. Further research should explore its impact on the salivary proteome and the resulting availability of calcium and phosphate, its potential role in preventing dental erosion, and how deficiency affects the composition and stability of the oral microbiome, particularly with respect to cariogenic bacteria. It should also be examined whether protective effects observed in primary teeth are replicated in permanent dentition, and whether any benefits result from higher salivary calcium levels or from the direct presence of vitamin D in saliva.

## Conclusion

Despite the availability of fluoride and improved oral hygiene, caries remains the most prevalent disease worldwide. More and more products are being developed, yet as early as 100 years ago, studies demonstrated that sufficient vitamin D intake could arrest carious lesions. The fact that vitamin D could play an important role in arresting existing lesions and the prevention of the formation of new lesions has been widely ignored in the modern past. Therefore, Vitamin D is experiencing a renaissance in current dental research. Further basic research is needed to clarify the potential mechanisms of action of vitamin D before longitudinal studies can evaluate its effects on the prevention and arrest of initial caries lesions in primary and permanent teeth.

## Data Availability

No datasets were generated or analysed during the current study.
